# Plasma gp96 is a Novel Predictive Biomarker for Severe COVID-19

**DOI:** 10.1128/Spectrum.00597-21

**Published:** 2021-11-24

**Authors:** Rongguo Wei, Biyan Zhou, Shaohua Li, Debin Zhong, Boan Li, Jianqiu Qin, Liping Zhao, Lixian Qin, Jun Hu, Jiuru Wang, Shixiong Yang, Jingming Zhao, Songdong Meng

**Affiliations:** a CAS Key Laboratory of Pathogenic Microbiology and Immunology, Center for Biosafety Mega-Science, Institute of Microbiology, Chinese Academy of Sciences, Beijing, China; b University of Chinese Academy of Sciences, Beijing, China; c Department of Clinical Laboratory, The Fifth Affiliated Hospital of Guangxi Medical University, Nanning, China; d Department of Pathology and Hepatology, The 5th Medical Centre, Chinese PLA General Hospital, Beijing, China; e Department of Clinical Laboratory, The 5th Medical Centre, Chinese PLA General Hospital, Beijing, China; f Nanning Municipal Center for Disease Control and Prevention, Nanning, China; University of Mississippi Medical Center

**Keywords:** plasma gp96, predictive biomarker, COVID‐19, IL-6

## Abstract

Early and effective identification of severe coronavirus disease 2019 (COVID-19) may allow us to improve the outcomes of associated severe acute respiratory illness with fever and respiratory symptoms. This study analyzed plasma concentrations of heat shock protein gp96 in nonsevere (including mild and typical) and severe (including severe and critical) patients with COVID-19 to evaluate its potential as a predictive and prognostic biomarker for disease severity. Plasma gp96 levels that were positively correlated with interleukin-6 (IL-6) levels were significantly elevated in COVID-19 patients admitted to the hospital but not in non-COVID-19 patients with less severe respiratory impairment. Meanwhile, significantly higher gp96 levels were observed in severe than nonsevere patients. Moreover, the continuous decline of plasma gp96 levels predicted disease remission and recovery, whereas its persistently high levels indicated poor prognosis in COVID-19 patients during hospitalization. Finally, monocytes were identified as the major IL-6 producers under exogenous gp96 stimulation. Our results demonstrate that plasma gp96 may be a useful predictive and prognostic biomarker for disease severity and outcome of COVID-19.

**IMPORTANCE** Early and effective identification of severe COVID-19 may allow us to improve the outcomes of associated severe acute respiratory illness with fever and respiratory symptoms. Some heat shock proteins (Hsps) are released during oxidative stress, cytotoxic injury, and viral infection and behave as danger-associated molecular patterns (DAMPs). This study analyzed plasma concentrations of Hsp gp96 in nonsevere and severe patients with COVID-19. Significantly higher plasma gp96 levels were observed in severe than those in nonsevere patients, and its persistently high levels indicated poor prognosis in COVID-19 patients. The results demonstrate that plasma gp96 may be a useful predictive and prognostic biomarker for disease severity and outcome of COVID-19.

## INTRODUCTION

The ongoing coronavirus disease 2019 (COVID-19) pandemic, caused by severe acute respiratory syndrome coronavirus 2 (SARS-CoV-2), has led to the infection of more than 170 million individuals and over 3.6 million deaths (as of 3 June 2021, from https://www.who.int/). In disease progression of COVID-19, excessive edema in the upper trachea and subsequent destruction of type I and II pneumocytes and alveolar/pulmonary edema are often observed as the main pathogenesis in the patient (1). The SARS-CoV-2 spike (S) protein recognizes and binds to angiotensin-converting enzyme-2 (ACE2) on type II alveolar cells, goblet cells, and ciliated cells in the airways, triggering the infection (2). SARS-CoV-2 replication in infected cells and release of virions could trigger a robust innate immune response through cellular pattern recognition receptors (PRRs), including Toll-like receptors (TLRs), retinoic acid–inducible gene-I, and melanoma differentiation-associated gene 5 (3). Besides mediation of viral entry, binding of SARS-CoV-2 to ACE2 dysregulates the renin-angiotensin system, leading to the inflammatory response. Angiotensin II (Ang II) binds with plasma membrane receptor AT1R and transduces signals to activate nuclear factor kappa B (NF-κB), signal transducer and activator of transcription 3, and other inflammatory transcription factors. This process significantly increases the level of proinflammatory cytokines, such as interleukin (IL)-1, IL-6 and tumor necrosis factor alpha (TNF-α). In addition, Ang II and AT1R interaction activates macrophages to produce excessive inflammatory cytokines that further result in a “cytokine storm” (4, 5). Furthermore, the infected cells may undergo apoptosis or necrosis that also initiates inflammatory responses (6). Excessive proinflammatory cytokines may cause tissue injury in multiple organs (e.g., heart, liver, and kidney). They also cause massive infiltration of neutrophils and macrophages in the lungs, leading to respiratory failure (5, 7). IL-6 has been shown to be the main driver of the cytokine storm and is associated with elevated levels of proinflammatory cytokines, leading to T cell depletion and pulmonary inflammation (6).

With skyrocketed COVID-19 cases, an urgent clinical need is to identify patients at higher risk of developing acute respiratory failure in the early stages of infection so that monitoring and interventions can be implemented in time to reduce the mortality of these patients. Currently, common laboratory indicators related to severe COVID-19 include biochemical (C-reactive protein, lactate dehydrogenase, troponins, and creatine kinase), hematologic (lymphocyte and neutrophil), coagulation (prothrombin times and d-dimer), and inflammatory parameters (8). Meanwhile, various prediction models are being reported to stratify the risk of COVID-19 patients. However, there are few biomarkers identified with high specificity for predicting severe COVID-19.

Some heat shock proteins (Hsps) are released during oxidative stress, heat shock, cytotoxic injury, and viral infection and behave as danger-associated molecular patterns (DAMPs) (9–11). Glucose-regulated protein gp96 (also known as GRP94) is one such Hsp. Under stress conditions, such as infection or inflammation, the intracellular levels of gp96 increase and lead to the activation of innate immune responses by stimulating the secretion of proinflammatory cytokines or chemokines via interaction with CD91 or TLRs on different immune cells (12–15).

The purpose of this study was to identify a useful indicator that can be used to guide treatment decisions to predict the severity and progression of COVID-19 disease. Therefore, we analyzed the plasma concentration of gp96 in hospitalized patients for the first time to evaluate its potential as a predictive and prognostic biomarker of COVID-19 disease severity.

## RESULTS

### Baseline characteristics.

Table 1 shows the demographics and baseline characteristics of the COVID-19 population. The patients were aged between 18 and 90 years old, with an average age of 52.4 years, and 19 of the 51 patients (37.3%) were over 60 years old. Of these patients, 22 were males, accounting for 43.1%, and 29 were females, accounting for 56.9%.

**TABLE 1:** Demographics and baseline characteristics of COVID-19 patients

Characteristic	Values for all patients (*n* = 51)	Values for patients by COVID type		*P* value^*a*^
		Nonsevere (*n* = 28)	Severe (*n* = 23)	
Median age (IQR^*b*^)	50 (40.5–67)	46.5 (26.75–57.5)	65 (46.5–72.5)	0.0014
Age subgroups (*n* [%])				
18–60 yrs	32 (62.7)	22 (78.6)	10 (43.5)	
>60 yrs	19 (37.3)	6 (21.4)	13 (56.5)	
Male (*n* [%])	22 (43.1)	10 (35.7)	12 (52.2)	0.27
Signs and symptoms (*n* [%])				
Fever	43 (84.3)	22 (78.6)	21 (91.3)	0.27
Cough	31 (60.8)	11 (39.3)	20 (87.0)	0.0006
Dyspnoea	16 (31.4)	2 (7.1)	14 (60.9)	<0.0001
Sputum production	10 (19.6)	3 (10.7)	7 (30.4)	0.15
Headache	14 (27.5)	9 (32.1)	5 (21.7)	0.53
Myalgia or fatigue	19 (37.3)	8 (28.6)	11 (47.8)	0.24
Comorbidity	31 (60.8)	14 (50.0)	17 (73.9)	0.09
Respiratory system disease	15 (29.4)	6 (21.4)	9 (39.1)	0.22
Diabetes	8 (15.7)	2 (7.14)	6 (26.1)	0.12
Hypertension	10 (19.6)	3 (10.7)	7 (30.4)	0.15
Laboratory characteristics (median [IQR])				
White blood cell count (10^9^/liter)	5.60 (4.04–6.90)	4.84 (3.86–6.56)	6.47 (4.87–8.40)	0.0269
Hemoglobulin (g/liter)	123 (112-133)	125 (119–140)	115 (98–125)	0.0075
Platelet count (10^9^/liter)	221 (171–264)	221 (149–259)	246 (176–290)	0.21
Neutrophile count (10^9^/liter)	3.51 (2.60–5.40)	2.80 (2.36–3.73)	4.81 (2.96–6.30)	0.0045
Lymphocyte count (10^9^/liter)	1.11 (0.66–1.59)	1.39 (0.86–1.74)	0.91 (0.49–1.13)	0.0007
C-reactive protein (mg/liter)	7.60 (2.44–19.70)	5.00 (2.90–9.36)	12.69 (2.04–36.40)	0.0408
Procalcitonin (ng/mL)	0.049 (0.033–0.072)	0.052 (0.038–0.066)	0.045 (0.031–0.085)	0.65
NT-pro brain natriuretic peptide (pg/mL)	85.41 (41.31–384.5)	29.47 (11.13–110.1)	208.2 (83.86–694.6)	0.12
Hypersensitive troponin T (pg/mL)	0.005 (0.003–0.010)	0.004 (0.003–0.004)	0.008 (0.004–0.022)	0.18
CD3^+^ cell count (cell/μL)	691 (402–1036)	955 (534–1376)	425 (277–715)	0.0006
CD4^+^ cell count (cell/μL)	356 (222–520)	430 (260–701)	246 (181–365)	0.0042
CD8^+^ cell count (cell/μL)	241 (167–492)	346 (235–583)	184 (89–292)	0.0076
CD4^+^/CD8^+^	1.25 (0.92–1.99)	1.22 (0.90–1.78)	1.45 (0.96-2.22)	0.91
Alanine aminotranferase (U/liter)	21.75 (14.75–34.00)	20.15 (13.00–32.75)	26.00 (18.75–37.88)	0.0387
Aspartate transaminase (U/liter)	24 (19–33)	23.5 (19–32.4)	29 (18.8–49.3)	0.0228
Total bilirubin (μmol/liter)	7.90 (6.80–11.10)	7.60 (6.62–9.40)	11.0 (7.28–15.23)	0.0037
Direct bilirubin (μmol/liter)	3.07 (2.10–4.70)	2.60 (1.96–3.18)	4.05 (2.90–6.33)	0.0113
Total protein (g/liter)	64.5 (58.0–74.3)	68.0 (62.7–77.0)	63.0 (57.0–72.0)	0.0949
Globulin (g/liter)	27.0 (24.9–32.0)	26.4 (24.2–31.0)	29.4 (24.9–37.6)	0.0336
Albumin globulin ratio	1.40 (1.08–1.66)	1.61 (1.36–1.83)	1.07 (0.85–1.41)	<0.0001
Potassium (mmol/liter)	4.0 (3.8–4.5)	4.0 (3.8–4.3)	4.0 (3.8–4.5)	0.76
Sodium (mmol/liter)	138.5 (136.0–141.0)	139.0 (138.0–141.0)	138.0 (136–141.0)	0.62
Glucose (mmol/liter)	5.8 (4.9–7.0)	5.3 (4.7–6.3)	6.5 (5.3–7.9)	0.0287
Urea (mmol/liter)	4.6 (3.8–6.1)	4.4 (3.8–5.0)	5.7 (3.9–6.7)	0.0206
Creatinine (μmol/liter)	68.5 (59.0–80.9)	67.9 (58.0–81.5)	69.9 (60.0–80.9)	0.36
d-dimer (μg/mL)	0.33 (0.19–0.65)	0.22 (0.14–0.33)	0.75 (0.33–5.01)	0.0019
Fibrinogen (g/liter)	3.01 (2.06–3.78)	3.41 (2.86–3.96)	2.61 (1.43–3.61)	0.0367
Activated partial thromboplastin time(s)	33.3 (28.0–38.0)	33.2 (29.5–36.6)	33.7 (25.6–46.0)	0.25
Prothrombin time (s)	12.9 (12.4–13.8)	12.9 (12.3–13.4)	13.5 (12.5–15.5)	0.0568
Creatine kinase (U/liter)	60.0 (41.0–103.8)	60.0 (46.3–84.8)	60.0 (36.5–127.3)	0.28
Lactic dehydrogenase (U/liter)	210.7 (164.3–248.3)	176.5 (159.0–207.6)	247.0 (233.3–298.0)	0.0006

a *P* values indicate differences between nonsevere and severe patients. *P* values of <0.05 were considered statistically significant.

b IQR, interquartile range.

At the time of admission, 43 (84.3%) patients had fever and 31 (60.8%) presented with cough. Other uncommon symptoms included dyspnea (31.4%), sputum production (19.6%), headache (27.5%), and myalgia or fatigue (37.3%). Most symptom profiles were comparable between the nonsevere group (including mild and typical; *n* = 28) and the severe group (including severe and critical; *n* = 23), while cough and dyspnea occurred in a significantly higher proportion of severe patients (*P* = 0.0006 for cough and *P* < 0.0001 for dyspnea). Moreover, respiratory system disease (15 [29.4%]), diabetes (8 [15.7%]), and hypertension (10 [19.6%]) were the most common coexisting conditions.

We found numerous differences in laboratory findings between the nonsevere and severe patients, including the white blood cell count, hemoglobin, neutrophil count, lymphocyte count, C-reactive protein, CD3^+^ cell count, CD4^+^ cell count, CD8^+^ cell count, alanine aminotransferase, aspartate transaminase, total bilirubin, direct bilirubin, globulin, albumin globulin ratio, d-dimer, fibrinogen, and lactic dehydrogenase. Levels of platelet count, procalcitonin, N-terminal pro brain natriuretic peptide, hypersensitive troponin T, total protein, potassium, sodium, creatinine, activated partial thromboplastin time, prothrombin time, and creatine kinase did not differ between the two groups (Table 1).

### Plasma gp96 levels correlate with IL-6 and COVID-19 severity.

**FIG 1:**
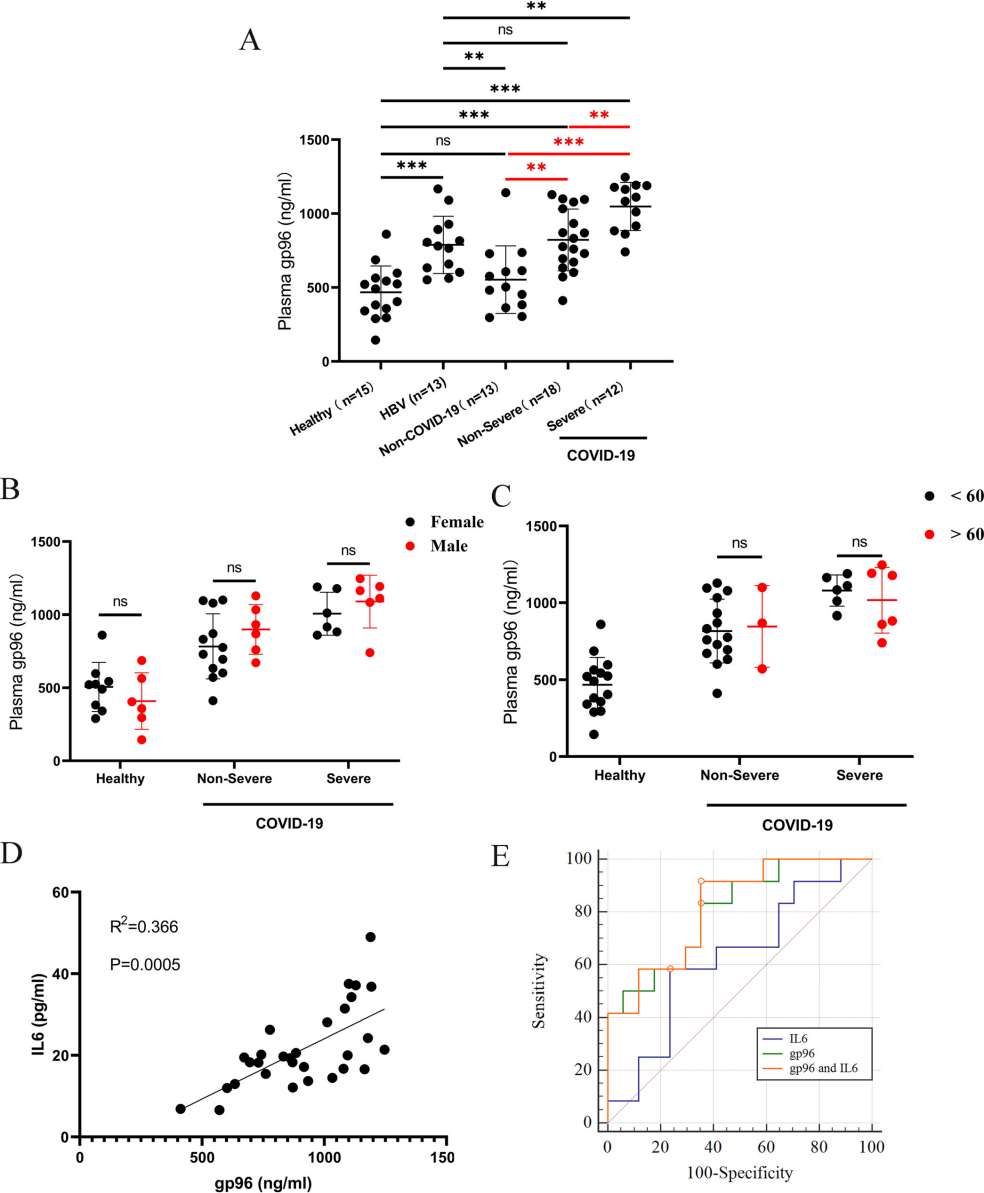
Plasma gp96 levels and ROC curve in COVID-19 patients on admission. (A) Comparison of plasma gp96 concentrations in COVID-19 patients, non-COVID-19 patients, HBV-infected patients, and healthy controls. (B and C) Comparison of plasma gp96 concentrations by age (<60 and >60 year) and sex (female and male) subgroups of COVID-19 patients and of healthy controls. (D) Correlation analysis between IL-6 and gp96 levels in patients with COVID-19. (E) ROC analysis of plasma gp96 and IL-6 concentrations between severe and nonsevere COVID-19 patients. Data are presented as mean ± SD. ns, not significant; **, *P* < 0.01; ***, *P *< 0.001.

We first conducted a cohort study of 12 severe COVID-19 patients, 18 nonsevere COVID-19 patients, 13 non-COVID-19 patients, 13 hepatitis B virus (HBV)-infected patients as non-SARS-CoV-2 virus infection controls, and 15 healthy controls. According to the results shown in Fig. 1A, we found that gp96 concentrations in the plasma increased significantly in all COVID-19 patients (912.7 ± 219.7), compared with those observed in healthy controls (467.5 ± 178.6). Both nonsevere (822.2 ± 208.8) and severe (1,048.0 ± 162.4) COVID-19 patients exhibited higher plasma gp96 levels than non-COVID-19 patients (553.4 ± 228.7) (*P *< 0.002). Furthermore, among COVID-19 patients, the severe group had significantly higher gp96 levels than the nonsevere group (*P *= 0.0037). No difference of plasma gp96 was observed between the non-COVID-19 group and healthy controls (*P *> 0.05). Meanwhile, the HBV-infected group also displayed higher plasma gp96 levels (788.8 ± 193.4) than the non-COVID-19 group (*P* = 0.0092) but much lower levels than the severe COVID-19 group (*P* = 0.0014). There was no significant difference between nonsevere COVID-19 and HBV-infected patients (*P *> 0.05).

We further conducted a subgroup analysis for age and sex for COVID-19 patients and healthy individuals to evaluate the impact of age and sex as the covariates. As can been seen in Fig. 1B and C, there were no significant differences of plasma gp96 between age subgroups <60 and >60 years in COVID-19 individuals, as well as the female and male subgroups within COVID-19 and healthy groups. Similar results were observed for plasma IL-6 (see Fig. S1 in the supplemental material).

The precise association between plasma gp96 and IL-6 levels was analyzed using linear regression models. The results showed that gp96 concentration was positively correlated with that of IL-6 (r^2^ = 0.366, *P* = 0.0005) (Fig. 1D).

To evaluate and compare the prognostic value of single and joint risk factors for gp96 and IL-6, receiver operating characteristic (ROC) was analyzed to calculate the area under the concentration-time curve (AUC) and corresponding specificity and sensitivity, with nonsevere type as the negative and severe type as the positive (Fig. 1E). The results showed that the AUC of gp96 was 0.815 (95% confidence interval [CI], 0.631 to 0.932; *P *< 0.001) and the best cutoff value was 871.21 ng/mL. Moreover, plasma gp96 showed a sensitivity of 83.3% and a specificity of 66.7% for differentiating severe from nonsevere patients. Compared with gp96, IL-6 alone showed slightly weaker prediction of severe COVID-19 with an AUC of 0.792 (95% CI, 0.605 to 0.917; *P *< 0.05). The combination of gp96 and IL-6 improved the predictive power, and the AUC, sensitivity, and specificity were 0.819 (95% CI, 0.637 to 0.935), 83.3% and 72.2%, respectively.

### Plasma gp96 predicts disease outcome.

**FIG 2:**
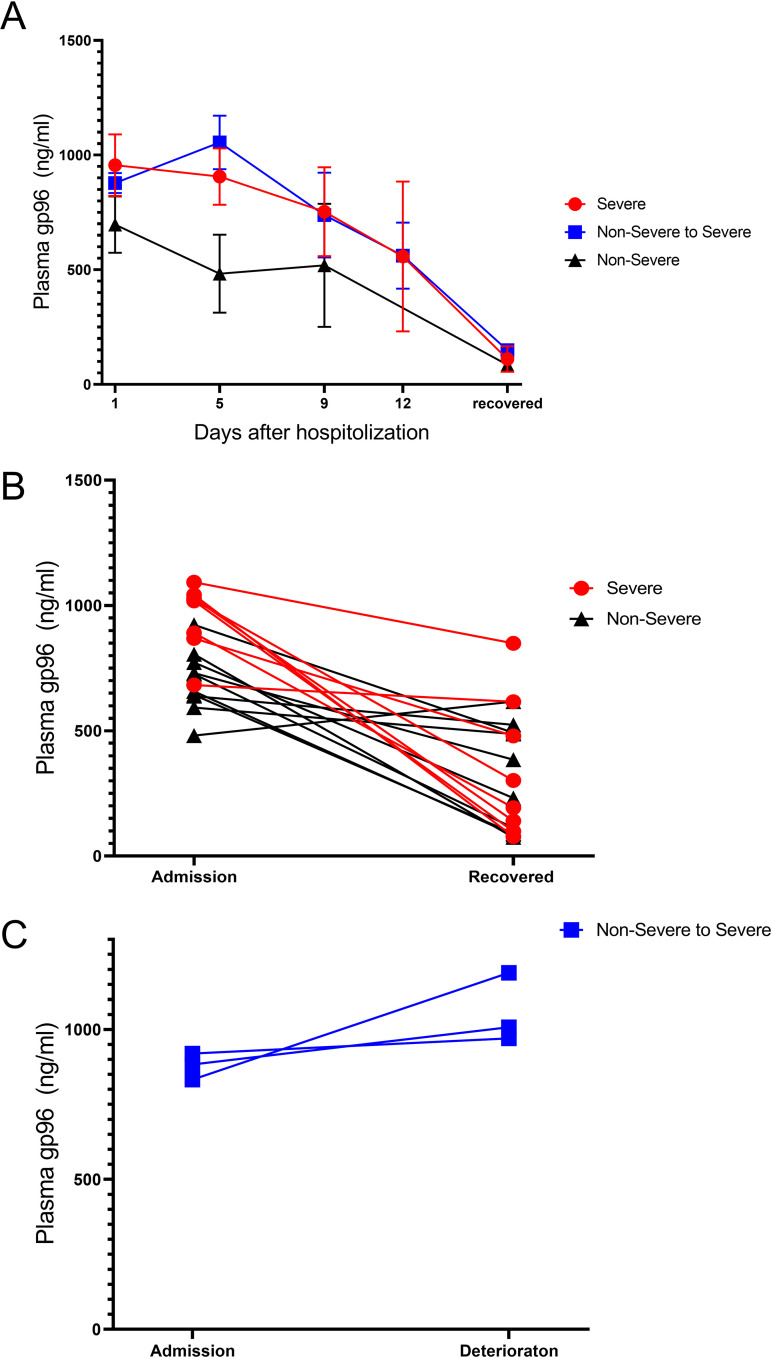
Dynamic characteristics of plasma gp96 levels in COVID-19 patients with disease recovery or deterioration. (A) Time course of plasma gp96 changes in severe (*n* = 11) and nonsevere patients (*n* = 10) during discovery. Data are represented as the median (IQR). A generalized linear mixed model was used to compare repeated measures (nonnormal distribution). (B) Changes in plasma gp96 levels of recovered COVID-19 patients between the time of admission and hospital discharge. (C) Changes in plasma gp96 levels in patients with disease deterioration. Data are the mean of triplicate measurements.

Next, we monitored dynamic changes of plasma gp96 in another cohort group with 11 severe and 10 nonsevere COVID-19 patients. From the time point at admission to the time point at the treatment endpoint, patients with continuous decline in plasma gp96 levels were more likely to have improved conditions and recovery (696.9 ± 123.0 to 127.3 ± 71.11 for nonsevere; 934.8 ± 119.7 to 303.6 ± 250.1 for severe) (Fig. 2A and B). A total of three exacerbating events occurred during hospitalization, which were observed among patients with gp96 levels of >832.63 ng/mL on admission. Nonsevere patients with increased plasma gp96 levels were more likely to develop severe COVID-19 (Fig. 2C).

### Exogenous gp96 stimulation led to an increase in IL-6 production by monocytes.

**FIG 3:**
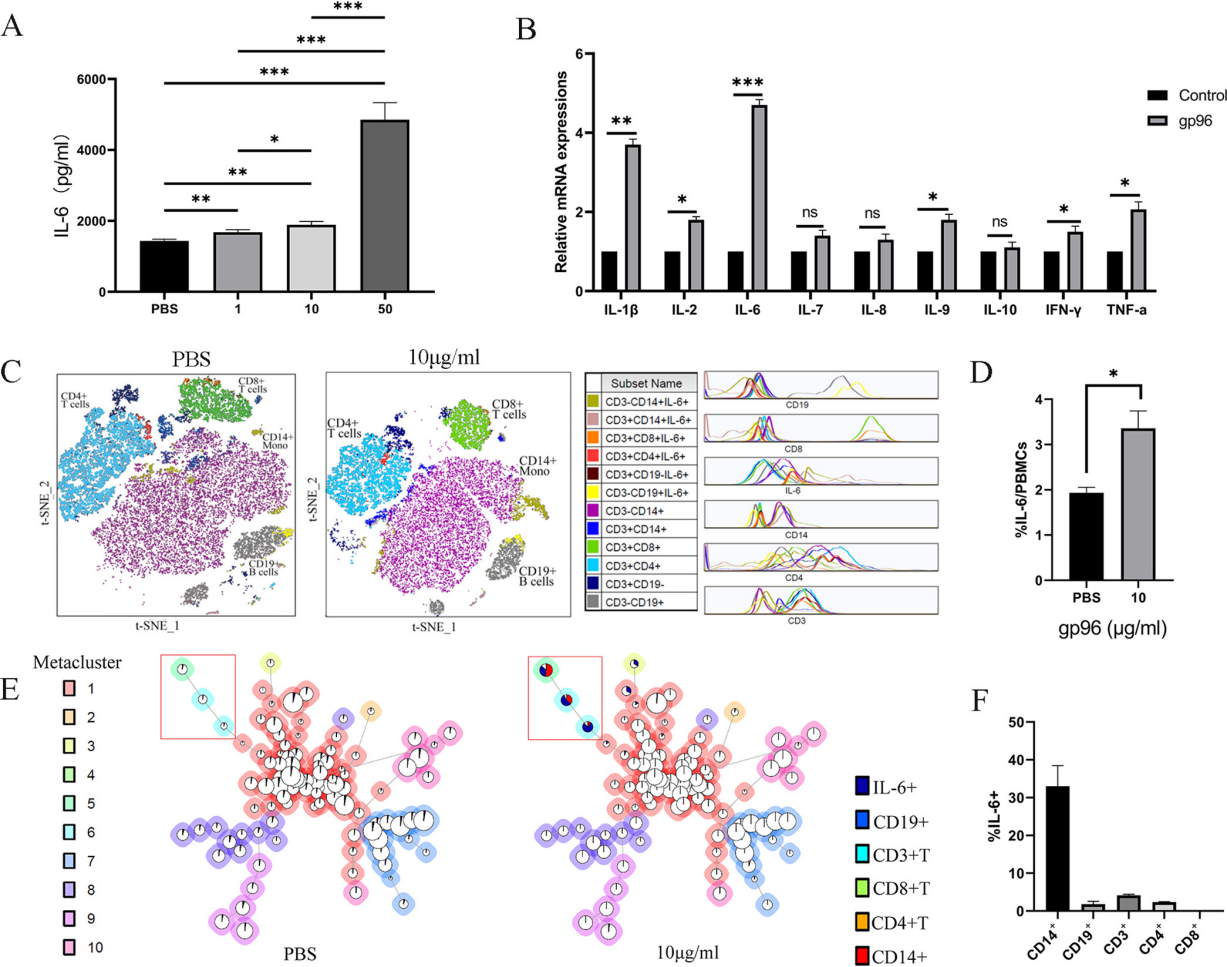
gp96 induces the expression of inflammatory cytokines of PBMCs *in vitro*. (A) PBMCs were incubated with the indicated doses of gp96 protein. At 24 h after treatment, secreted IL-6 was detected in the culture supernatant using ELISA. (B) PBMCs were treated with 10 μg/ml gp96 or PBS (control) for 12 h. The relative mRNA expression of multiple inflammatory cytokines was determined by quantitative real-time PCR. (C) PBMC subtypes visualized by t-SNE plot. PBMCs were treated with 10 μg/ml gp96 for 24 h. Using the t-SNE algorithm, cellular subsets from a sample were clustered into immune phenotype with 50,000 cells per leukocyte population based on similarities in expression profiles of individual cells. This procedure successfully clustered populations by the differential expression of lineages. (D) Percentage of IL-6^+^ cells in PBMCs treated with PBS and 10 μg/ml gp96. (E) FlowSOM results from one representative sample treated with PBS or 10 μg/ml as minimum spanning trees. FlowSOM was performed using 225 clusters and 10 metaclusters. Each cluster is represented by 1 pie chart, and metaclusters are denoted by background shading. (F) Percentage of CD14^+^, CD19^+^, CD3^+^, CD3^+^CD4^+^, and CD3^+^CD8^+^ T cells among IL-6^+^ cells in PBMCs from healthy donors treated with 10 μg/ml gp96. Data are presented as mean ± SD from three independent experiments. ns, not significant; *, *P *< 0.05; **, *P *< 0.01; ***, *P *< 0.001.

Finally, we determined if exogenous gp96 could act as a DAMP molecule to stimulate IL-6 secretion by immune cells. As seen in Fig. 3A, an enzyme-linked immunosorbent assay (ELISA) analysis revealed that gp96 induced IL-6 secretion in a dose-dependent manner. The expression of key inflammatory cytokines in peripheral blood mononuclear cells (PBMCs), including IL-1β, IL-2, IL-6, interferon gamma (IFN-γ), IL-9, and TNF-α, were considerably increased under gp96 stimulation *in vitro* (Fig. 3B). Furthermore, significantly increased IL-6-secreting immune cells were observed by gp96 treatment (Fig. 3C and D). In addition, a moderate increase of IL-6 was also observed in gp96-treated PBMCs by Western blotting (see Fig. S2 in the supplemental material). No dose-dependent effect by gp96 was seen for cellular IL-6 levels. This finding may be due to rapid IL-6 precursor protein processing and secretion of mature IL-6 to outside cells.

Meanwhile, PBMCs were treated with 10 μg/mL gp96 for 24 h, and the cells were stained with markers of T cells, B cells, and monocytes and then analyzed by flow cytometry. We further used t-distributed stochastic neighbor embedding (t-SNE) and the FlowSOM algorithm to analyze and visualize the results (see Fig. S3 in the supplemental material). As shown in Fig. 3C, both the gp96-treated and control (phosphate-buffered saline [PBS]) group exhibited similar immune cell frequencies, with predominant CD14^+^ cells, followed by CD3^+^CD4^+^ cells and CD3^+^CD8^+^ cells, and CD19^+^ cells had the smallest frequency. This result indicated that gp96 treatment did not affect the frequencies of T cells, B cells, and monocytes. But, the percentage of IL-6^+^ cells in PBMCs treated with gp96 was significantly higher than that of the control group (*P *< 0.05) (Fig. 3D).

To validate our findings, we further used FlowSOM to cluster, visualize, and compare the data from the IL-6^+^ cell gate of a 6-color flow cytometry panel. FlowSOM is based on self-organizing map (SOM) to analyze flow cytometry data, which is an algorithm that can be used to analyze flow cytometry and high-dimensional data. FlowSOM helps to understand all the marked features clearly and comprehensively in all cells and can detect subsets that are easily ignored by other algorithms. Cells of the same phenotype were clustered into populations with the algorithm. Each node representing one population was presented as a colored metacluster on the minimum spanning tree (16). Using FlowSOM, we conducted a comparative statistical analysis of the proportions of each subset. The results revealed an increase in CD14^+^IL-6^+^ cells, as shown in the red frame, in the gp96-treated group compared with the control group (Fig. 3E). Under gp96 stimulation, IL-6 was produced mainly from CD14^+^ monocytes (33.03% ± 5.45%), followed by CD3^+^ T cells (4.14% ± 0.30%) and CD19^+^ B cells (1.82% ± 0.75%) (Fig. 3F). The results show that by profiling the IL-6 expression status in different types of immune cells, monocytes were identified as the major IL-6 producer. These results suggest that extracellular gp96 may act as a proinflammatory mediator in immune hyperactivation during COVID-19 infection.

## DISCUSSION

In this study, extracellular gp96 was analyzed for the first time in the blood of patients with COVID-19. Plasma concentrations of gp96 were significantly elevated in both nonsevere and severe COVID-19 patients admitted to the hospital compared with those of healthy controls, but not in non-COVID-19 patients with less severe respiratory impairment. Although these results are preliminary with limitations in selection bias, a small number of patients, and use of a single parameter, they are of interest because gp96 appears to be a potential predictive and prognostic biomarker that is easily detectable in the blood of patients. The development of such a biomarker would help to phenotype patients according to the severity and outcome of the disease.

Aberrant expression of gp96 has been reported to be associated with vesicular stomatitis virus (VSV), rotavirus, Japanese encephalitis virus (JEV), and HBV infections (17). The ubiquitous expression of gp96 is considered essential in VSV infection (18). A study on JEV has shown that extracellular gp96 is released into the secretory medium after JEV infection (19). HBV chronically infected patients show high levels of gp96 expression in the liver, which is related to poor prognosis (20, 21). In the current study, we also found the mean plasma gp96 level of HBV-infected patients was 1.69 times higher than that in the healthy controls. The gp96 level of the HBV-infected group was not significantly different from that of nonsevere COVID-19 patients but was lower than that of severe COVID-19 patients (see Fig. 1A). These results show that elevated gp96 in plasma was not COVID-19 specific since several other viral infections have been shown to lead to increased gp96 levels. Therefore, unlike real-time PCR-based assays that are the current gold standard for confirmed infection, plasma gp96 cannot be used solely for SARS-CoV-2 diagnosis. The ROC curve analysis revealed significant prognostic value of plasma gp96 for predicting severe COVID-19. When plasma IL-6 and gp96 were jointly predicted, the ROC curve integral of severe COVID‐19 was increased to 0.819, which was a good predictor of severe COVID-19. This finding indicates that combined detection was more efficient than independent detection. Hence, during the hospitalizations of COVID-19 patients, monitoring plasma or serum gp96 and IL-6 levels has a certain value and better effectiveness in early warnings of disease severity. Hematological indicators, such as C‐reactive protein, d‐dimer, fibrinogen, and thrombin time were often monitored in clinical practice. Joint monitoring with gp96 can play an important complementary role. Choosing different combinations according to different situations can highly strengthen the accuracy of the prediction of severe COVID-19. In addition, it is possible that a dual viral infection may result in more elevated plasma gp96 levels. This possibility deserves further investigation.

Aging is related to patient susceptibility to infection by certain viruses (22). For COVID-19, previous studies have suggested that older patients have an increased risk of progression to severe disease, adult respiratory distress syndrome, and mortality (23). Other studies have shown that males are significantly associated with increased mortality from COVID-19 relative to females (24). Our study also found that the age of patients with severe COVID-19 was significantly higher than that of nonsevere patients (*P *< 0.05), and the percentage of patients aged >60 years in the severe group was 2.64 times higher than that of the nonsevere group. However, in the subgroup statistics of patients aged <60 and >60 years, we found no significant differences of plasma gp96 and IL-6 levels between these two groups. It is possible that the COVID-19 targeted organs in elder individuals are more fragile to inflammation-induced pathogenesis.

Our results (Fig. 1D) show that plasma gp96 and IL-6 levels were weakly correlated in COVID-19 patients, while gp96 was found to be a strong inducer of IL-6 *in vitro* (Fig. 3A and B). Two possible reasons may explain this discrepancy. First, besides plasma gp96, viral RNA and multiple other elevated DAMPs could also induce IL-6 expression, including cell-free DNA, extracellular histone H3, and neutrophil elastase, as well as the immune modulators GAS6 and AXL (11, 25). In addition, in severe COVID-19, the levels of circulating mitochondrial DNA, S100A8/A9, and high mobility group box 1 were also significantly increased and were correlated with inferior clinical outcomes (11, 26). Second, it has been found that after SARS-CoV-2 infection, the cytokine production function and chemotaxis of blood monocytes are disrupted (27), which to some extent may lead to a weak correlation between gp96 and IL-6. More studies are needed to dissect the precise mechanisms of plasma gp96 as an upstream proinflammatory inducer in the context of other DAMPs and different immune cell statuses in COVID-19.

In this study, the mechanism of the proinflammatory potential of gp96 was explored in COVID-19. The results show that coincubation of gp96 with PBMCs *in vitro* promoted IL-6 secretion that was primarily produced by CD14^+^ monocytes (Fig. 3F). In the process of SARS-CoV-2 infection, monocytes and macrophages were shown to participate in hypersensitivity and exacerbation reactions that result in lung injury, and monocytes may be a potential source of inflammatory cytokines, including IL-6, which are closely related to the inflammatory phenotype of severe COVID-19 (27, 28). Single-cell RNA sequencing of blood antigen-presenting cells in severe COVID-19 reveals that pathways related to NF-κB signaling were upregulated in plasmacytoid dendritic cells and CD14^+^ monocytes (29). We and others also show that extracellular gp96 induced proinflammatory cytokines (e.g., IL-6 and TNF-α) in activated immune cells (30–32). Taken together, our study indicates that extracellular gp96 is released from the damaged and necrotic cells after SARS-CoV-2 infection and stimulates monocytes and other proinflammatory immune cells to secrete IL-6 through the NF-KB pathway. This process may contribute to the progression of severe COVID-19.

Inflammatory activation plays a crucial role in the natural course of SARS-CoV-2 infection. SARS-CoV-2 infection may lead to an uncontrollable immune response and subsequent sepsis or septic shock through the release of various DAMPs and cytokines (33, 34). A number of studies have demonstrated the ability of extracellular gp96 as a DAMP to drive inflammation (30, 31). Our study found that levels of plasma gp96 and IL-6 were closely correlated in COVID-19 patients that and gp96 can act as DAMP to activate CD14^+^ cells to secrete IL-6. Therefore, compared with IL-6, elevated plasma gp96 from damaged or necrotic host cells by SARS-CoV-2 infection could act as an earlier indicator for pathogenesis and disease progression. This knowledge will allow medical intervention at earlier stages of disease deterioration. In addition, the ROC results show that the AUC area of gp96 is even relatively higher than that of IL-6. Conceivably, the combination of gp96 and IL-6, alongside thorough clinical assessment of patients, will enable more accurate stratification of high from low risk cases toward severe COVID-19. Our results therefore may reveal the underlying mechanism of extracellular gp96-derived excessive inflammation and provide plasma gp96 as a potential biomarker predicting the severity of disease progression in COVID-19.

In conclusion, elevated levels of plasma gp96 correlate with disease severity of COVID-19 patients, supporting its potential usefulness as an inflammatory biomarker for predicting outcome for severe COVID-19 patients.

## MATERIALS AND METHODS

### Ethical statement.

This study was approved by the Ethics Committee of the 5th Medical Centre, Chinese PLA General Hospital (approval no. GH-5-20200811; approval date, 11 August 2020).

### Study design and population.

Our team performed two cohort studies and procured plasma samples from 51 COVID-19 patients, 13 non-COVID-19 patients, 13 patients infected with HBV, and 15 healthy volunteers. The COVID-19 patients (median age [interquartile range {IQR}], 50 [40.5 to 67] years; 22 men) hospitalized at the Fifth Medical Center of PLA General Hospital from January to March 2020 were enrolled prospectively. Patients with a confirmed diagnosis of COVID-19 were divided into mild, typical, severe, and critical groups, according to the Chinese Government Diagnosis and Treatment Guideline (trial 6th version) (NHCPRC, 2020). In this study, we included both nonsevere and severe patients, with the former comprising patients with mild and typical COVID-19. All COVID-19 patients were not infected with other common viral, such as influenza virus, respiratory syncytial virus, HBV, human immunodeficiency virus, VSV, and adenovirus.

We recruited 13 non-COVID-19 patients (median age [IQR], 30 [24 to 31] years; 8 men) with similar clinical characteristics as COVID-19 patients, including 4 with pneumonia with fever, 6 fever, and 2 cough (see Table S1 in the supplemental material). Their nucleic acid testing for SARS-CoV-2 was all negative. We also enrolled 13 HBV-infected patients (median age [IQR], 37 [30 to 42] years; 6 men) with positivity for serum hepatitis B surface antigen by chemiluminescence (see Table S2 in the supplemental material) and 15 healthy volunteers (median age [IQR], 32 [30.5 to 35.5] years; 8 men) as a control group, with no history of concomitant pathologies and without taking any medication. All medical data of the patients were followed up, including epidemiological, demographic, laboratory, and clinical data and treatment outcomes.

### Plasma gp96 assay.

Plasma gp96 was measured using ELISA, as reported previously (30). The principle of the assay is agglutination of the carbohydrate antigen in samples with a gp96 monoclonal antibody by antigen-antibody reaction. The optical density (OD) value was measured to determine gp96 concentrations, which were expressed in ng/mL.

### *In vitro* assays.

The gp96 protein was purified from human placenta, as described by Zhao et al.(35). Before use of peripheral blood for *in vitro* assays and for flow cytometric analysis, PBMCs were isolated from 3 healthy donors using red blood cell lysis buffer (BD Biosciences). PBMCs were resuspended in RPMI 1640 (GIBCO) medium containing 10% heat-inactivated fetal bovine serum (Gibco), 25 μg/mL streptomycin (Gibco), and 100 IU/mL penicillin (Gibco), and aliquots were at a concentration of 1 × 10^6^ cells/mL/well in 6-well or 12-well culture plates (Corning). The cells were exposed to 0 to 50 μg/mL human gp96, and after 12 h or 24 h of culture, the cells and the suspension were harvested for ELISA (IL-6 human ELISA kit; Invitrogen), Western blot, real-time PCR, or flow cytometric analysis as described previously (30, 36). Western blotting was performed using mouse anti-GAPDH, rabbit anti-IL-6, goat anti-mouse IgG horseradish peroxidase (HRP), and goat anti-rabbit IgG HRP (Abcam). The primers used in this study are shown in Table S3 in the supplemental material. The flow cytometric staining antibodies included CD3 Alexa Fluor 700, CD4 PerCP-Cy5.5, CD8a PE, CD14 FITC, CD19 APC, and IL-6 PE-Cy7 (BD Biosciences). Samples were acquired on a FACSAria II (BD) instrument with FACS Diva version (BD; version 8.0.1) software. The analysis was completed using FlowJo (BD; version 10.6.2). We further used the t-distributed stochastic neighbor embedding (t-SNE) and FlowSOM algorithm to analyze and visualize the results. By using the t-SNE algorithm, cellular subsets from a sample were clustered into immune phenotype with 50,000 cells per leukocyte population based on similarities in expression profiles of individual cells. This process successfully clustered populations by the differential expression of lineages. For FlowSOM, the minimal spanning tree of a self-organizing map was visualized using flow cytometric data from the group treated with 10 μg/ml gp96 compared with the control (PBS) (both have equal number of events). Data were taken from the gated IL-6^+^ cells (16, 37).

### Statistical analysis.

The *t* test and independent sample chi-square test were used to analyze differences between the groups. Statistical significance was set at a *P* value of <0.05. The Spearman correlation coefficient was used to measure the degree of correlation between the hierarchically ordered variables in this study. The ROC curve analysis was performed on the sensitivity, specificity, and AUC of plasma gp96 and IL-6 using the MedCalc statistical program (version 19.0.7). Other statistical analyses and graphic representation of the data were performed using GraphPad Prism software (version 8.0).
