# Supplementary material for: Plasma gp96 is a Novel Predictive Biomarker for Severe COVID-19
Source: Microbiol Spectr. 2021 Nov 24;9(3):e00597-21. doi: 10.1128/Spectrum.00597-21 (PMC8612155; doi:10.1128/Spectrum.00597-21)
Supplement: SUPPLEMENTAL FILE 1 — Supplemental material. Download SPECTRUM00597-21_Supp_1_seq3.pdf, PDF file, 0.3 MB [file spectrum00597-21_supp_1_seq3.pdf]

Plasma gp96 is a novel predictive biomarker for severe COVID-19

Table S1. Non-COVID-19 patient details.

Patient	Age	Gender	CT scan	History of epidemiology	Clinical feature
101	24	female	/	/	fever
102	45	male	/	/	cough
103	20	male	/	/	fever
104	26	male	/	Wuhan	/
105	41	male	/	/	fever
106	18	male	/	/	fever
107	30	female	/	/	cough
108	21	male	/	Wuhan	fever
109	36	male	pneumonia	/	fever
110	31	female	/	Wuhan	fever
111	24	female	pneumonia	/	fever
112	31	female	pneumonia	Wuhan	fever
113	30	male	pneumonia	Wuhan	fever

Table S2. HBV-infected patient details.

Patient	Age	Gender	HBsAg	HBcAg	AFP (ng/mL)	ALT (U/L)
HBV01	50	female	+	-	2.08	239
HBV02	51	male	+	+	4.1	43
HBV03	42	male	+	-	/	114
HBV04	33	male	+	+	2.67	13
HBV05	26	female	+	-	2.9	14
HBV06	38	male	+	-	57.82	1322
HBV07	38	female	+	-	2.61	17
HBV08	28	female	+	-	1.88	14
HBV09	27	female	+	-	1.76	72
HBV10	34	female	+	-	/	156
HBV11	30	female	+	-	/	12
HBV12	37	male	+	-	2.1	19
HBV13	45	male	+	+	3.09	24

Table S3. Primers used in quantitative real-time PCR.

Gene name	Primer sequence 5`to 3`
IL1β F	ATGATGGCTTATTACAGTGGCAA
IL1β R	GTCGGAGATTCGTAGCTGGA
IL2 F	TACAAGAACCCGAAACTGACTCG
IL2 R	ACATGAAGGTAGTCTCACTGCC
IL6 F	ACTCACCTCTTCAGAACGAATTG
IL6 R	CCATCTTTGGAAGGTTCAGGTTG
IL7 F	GATCAAGATCATTGCTCCTCCT
IL7 R	AGGGTGTAAAACGCAGCTCA
IL8 F	TTTTGCCAAGGAGTGCTAAAGA
IL8 R	AACCCTCTGCACCCAGTTTTC
IL9 F	CTCTGTTTGGGCATTCCCTCT
IL9 R	GGGTATCTTGTTTGCATGGTGG
IL10 F	GACTTTAAGGGTTACCTGGGTTG
IL10 R	TCACATGCGCCTTGATGTCTG
IFN-γ F	TCGGTAACTGACTTGAATGTCCA
IFN-γ R	TCGCTTCCCTGTTTTAGCTGC
TNFα F	CCTCTCTCTAATCAGCCCTCTG
TNFα R	GAGGACCTGGGAGTAGATGAG

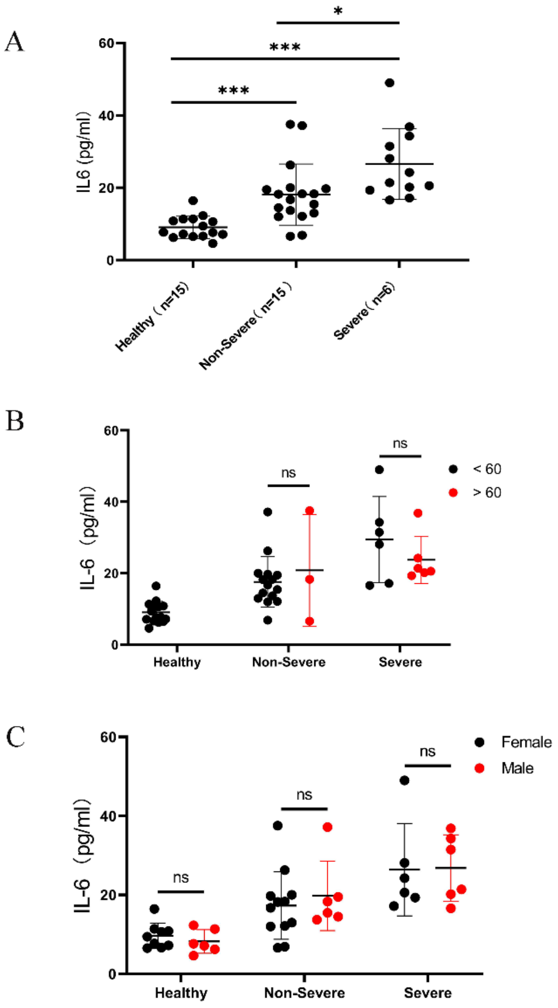

Fig. S1. Plasma IL6 levels in COVID-19 patients on admission. A, Comparison of IL6 concentrations in COVID-19 patients, and healthy controls. B-C, Comparison of IL6 concentrations in age (<60 and >60 yr) and gender (female and male) subgroups of COVID-19 patients, and healthy controls. Data are presented as mean ± SD. ns, not significant, *p < 0.05, **p < 0.01, and ***p < 0.001.

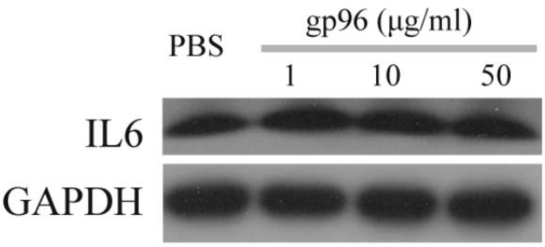

Fig. S2. A moderate increase of IL6 was observed in gp96-treated PBMCs by western blotting analysis. No dose-dependent effect by gp96 was seen for cellular IL6 levels. This may be due to rapid IL6 precursor protein processing and secretion of mature IL6 to outside cells.

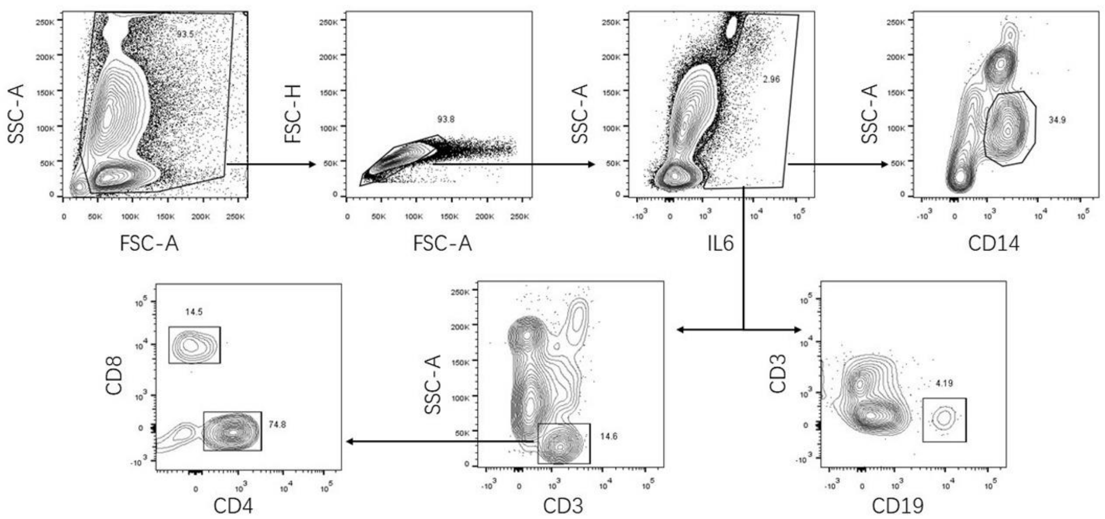

Fig. S3: Flow cytometric gating strategies. In all flow cytometric analyses, PBMC were identified by SSC and FSC properties. Identification of cells used in t-SNE and FlowSOM analysis gated sequentially as: cells, single cell, IL6+ cells, CD14+ cells, CD3-CD19+ cells, CD3+ cells, CD3+CD4+ cells and CD3+CD8+ cells.
